# Detecting Differential Transmissibilities That Affect the Size of Self-Limited Outbreaks

**DOI:** 10.1371/journal.ppat.1004452

**Published:** 2014-10-30

**Authors:** Seth Blumberg, Sebastian Funk, Juliet R. C. Pulliam

**Affiliations:** 1 Francis I. Proctor Foundation, University of California San Francisco, San Francisco, California, United States of America; 2 Fogarty International Center, National Institutes of Health, Bethesda, Maryland, United States of America; 3 Centre for the Mathematical Modelling of Infectious Diseases, London School of Hygiene & Tropical Medicine, London, United Kingdom; 4 Ecology and Evolutionary Biology, Princeton University, Princeton, New Jersey, United States of America; 5 Department of Biology, University of Florida, Gainesville, Florida, United States of America; 6 Emerging Pathogens Institute, University of Florida, Gainesville, Florida, United States of America; University of Texas at Austin, United States of America

## Abstract

Our ability to respond appropriately to infectious diseases is enhanced by identifying differences in the potential for transmitting infection between individuals. Here, we identify epidemiological traits of self-limited infections (i.e. infections with an effective reproduction number satisfying 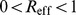) that correlate with transmissibility. Our analysis is based on a branching process model that permits statistical comparison of both the strength and heterogeneity of transmission for two distinct types of cases. Our approach provides insight into a variety of scenarios, including the transmission of Middle East Respiratory Syndrome Coronavirus (MERS-CoV) in the Arabian peninsula, measles in North America, pre-eradication smallpox in Europe, and human monkeypox in the Democratic Republic of the Congo. When applied to chain size data for MERS-CoV transmission before 2014, our method indicates that despite an apparent trend towards improved control, there is not enough statistical evidence to indicate that 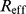 has declined with time. Meanwhile, chain size data for measles in the United States and Canada reveal statistically significant geographic variation in 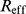, suggesting that the timing and coverage of national vaccination programs, as well as contact tracing procedures, may shape the size distribution of observed infection clusters. Infection source data for smallpox suggests that primary cases transmitted more than secondary cases, and provides a quantitative assessment of the effectiveness of control interventions. Human monkeypox, on the other hand, does not show evidence of differential transmission between animals in contact with humans, primary cases, or secondary cases, which assuages the concern that social mixing can amplify transmission by secondary cases. Lastly, we evaluate surveillance requirements for detecting a change in the human-to-human transmission of monkeypox since the cessation of cross-protective smallpox vaccination. Our studies lay the foundation for future investigations regarding how infection source, vaccination status or other putative transmissibility traits may affect self-limited transmission.

## Author Summary

The goal of this paper is to identify epidemiological factors that correlate with either an increased or decreased risk of transmitting a particular disease. We are particularly interested in identifying such factors for diseases that are *self-limited* (meaning that infections tend to occur in isolated clusters), because targeted control of these diseases can facilitate public health goals for minimizing the risk of disease emergence or promoting disease elimination. For example, we show that there is a significant difference in the transmission of measles between the United States and Canada. In contrast, we find that an observed decrease in the transmission of Middle East respiratory syndrome coronavirus during the latter half of 2013 cannot be ascertained with sufficient confidence. We then quantify the degree to which control was effective in eradicating smallpox in Europe. We also consider how the transmission of monkeypox in humans depends on whether the infection source is an animal or a human. Finally, we demonstrate how our approach can be used by surveillance programs to detect changes in transmission that may occur over time.

## Introduction

Many infections only occur as isolated cases, short chains of transmission, or as small infection clusters (i.e. intertwined transmission chains). Examples include zoonotic infections with relatively weak human-to-human transmission as well as vaccine-preventable infections in settings of high vaccination coverage [1]–[7]. Even though transmission is limited, these diseases are an important public health concern. For example, zoonotic infections can adapt for increased human-to-human transmission and then cause greater or even pandemic spread [8]–[10]. In addition, decreased voluntary vaccination, difficulty with vaccine delivery or changes in vaccine efficacy can allow growth of the number of individuals susceptible to preventable diseases and thus cause larger outbreaks [3], [11]. Self-limited (or *subcritical*) transmission also characterizes diseases that are on the brink of elimination such as smallpox during its worldwide eradication campaign or polio today [12]–[14].

Despite a need to monitor disease burden, manage the risk of disease emergence or enhance disease elimination, the surveillance and control of subcritical infections can be challenging. Resource-poor countries, which are home to many zoonoses, have many logistical hurdles that impact the quality of surveillance and control interventions. Meanwhile, even in developed countries, reactive control strategies such as isolation protocols for vaccine-preventable diseases have significant sociological impact beyond the immediate financial costs. Because of these challenges, the overarching goal is to optimize control interventions for the least amount of effort and expense. It is therefore important to gain as much quantitative information about disease transmission as possible from existing surveillance data. This includes monitoring how transmission varies with time, location and other epidemiological characteristics of individual cases. By improving the understanding of mechanisms of disease transmission, finer tuning within the spectrum of intervention strategies becomes possible [15], [16]. Such mechanistic understanding can guide the response to a diverse range of threats that include emerging infections (e.g., Middle East respiratory syndrome coronavirus), vaccine-preventable infections (e.g., measles) and antibiotic resistance [17], [18].

For ethical and logistical reasons, population-level studies of infectious disease transmission in humans typically involve retrospective statistical analysis rather than controlled prospective experimentation. Given this constraint, one approach for evaluating mechanisms underlying transmission patterns is to compare the transmissibility of two distinct, but related populations. In this manuscript, we demonstrate how the strength and heterogeneity of transmission can be compared for two different populations or types of infection sources. We then show how our framework provides insight into the transmission patterns of a variety of subcritical diseases. This analysis builds upon earlier studies that were limited to estimating transmission parameters from chain size distributions and addressing issues of surveillance bias [19], [20].

Mathematically, the transmissibility of a group of infected individuals can be quantified by determining the group's effective reproduction number, 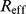. This number represents the mean number of secondary cases caused by an infected case. However, because of the stochastic nature of disease transmission, the realized numbers of secondary infections caused by a given infected individual will vary. 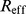 is a more general parameter than the oft cited *basic* reproduction number 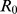, which more specifically represents the mean number of secondary cases caused by the first infected case in a completely susceptible population [21]. When 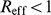, transmission cannot reach epidemic proportions, whereas if 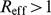 there is a potential for epidemic spread. Thus, our focus on subcritical diseases implies that, overall, 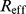 will be less than one and transmission will be characterized by self-limited clusters of infection. However, our method still permits the possibility that cases can be divided into two groups in which one group has a 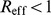, and the other group has a 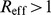.

Our study builds upon the prior success of inferring 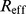 from the size distribution of observed transmission chains [1], [2], [22]. The same distributions can also be used to infer the degree of transmission heterogeneity, represented by the dispersion parameter, 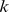 [19], [20], [23]. A high degree of heterogeneity represents a scenario where some individuals are predisposed to spreading infection to a larger number of people (i.e., ‘superspreaders’). When models of chain size distributions incorporate both 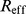 and 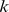, excellent agreement can often be found between observed data and model predictions [19], [20], [23].

Our goal is to evaluate specific hypotheses regarding disease transmission by testing whether 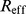 and 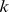 differ between two groups of cases. Our analyses differ from more traditional epidemiological approaches based on case-control studies (and many other study designs) in that we focus on transmissibility instead of individual-level risk factors for disease susceptibility. We demonstrate our methodology by considering four subcritical infections (MERS-CoV, measles, monkeypox and smallpox) and three types of data (size distribution of infection clusters, transmission chain data and infection source classification) to answer four different questions based on published data. For MERS-CoV, we use chain size distributions to determine whether an apparent decrease in 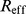 during the latter half of 2013 was statistically significant. Assessing temporal trends of 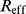 has important implications for evaluating the risk of endemic MERS-CoV transmission and the impact of control interventions. For measles, we use chain size distributions to compare two locations (United States and Canada) and test whether there is a significant difference in 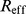, which would suggest important differences in vaccine distribution, social connectedness, and/or demographics. For smallpox and monkeypox, we use case series resolved by infection generation to determine whether there are significant differences between the first and subsequent generations of spread [24], [25]. This analysis allows us to assess whether variation in the number of contacts or the timing of control interventions can be linked to changes in 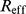. It also allows us to test the validity of a specific ‘random network’ model that relates the contact patterns of primary and secondary cases. We then test whether there is a significant difference between inferred transmission parameters for animal-to-human and human-to-human transmission of monkeypox, which provides insight into the mechanisms of zoonotic spillover. Our analysis of chain size distributions also provides perspective on the surveillance required to detect a change in 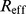, such as the expected increase in human monkeypox transmission following the eradication of smallpox. Each of the scenarios considered represents a unique example of how quantitative characterization of transmissibility can provide insight into the effectiveness of control interventions and risk assessment for future spread.

## Methods

### Modeling framework

The stochastic nature of infectious disease transmission is particularly important when 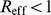, as it can result in substantial variation in the size distribution of transmission chains. In this case it is helpful to model transmission as a branching process [26]. In this formulation, the offspring distribution specifies the probability that an infected individual will cause 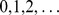 new infections. We specify the corresponding offspring probabilities to be 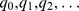, with 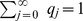. To facilitate likelihood calculations (as seen below), the offspring distribution can be represented as a generating function, 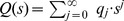, in which the polynomial coefficients are the offspring probabilities [26]–[28].

In line with research demonstrating how the strength and variability of transmission can be modeled [23], we assume the *q_i_*'s follow a negative binomial offspring distribution with a mean of 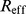 and a dispersion parameter of 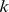. The dispersion parameter represents the degree of transmission heterogeneity, with lower values of 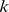 corresponding to higher variance. The supplementary methods (Text S1) explains how our simple model of disease transmission can be used to calculate the likelihood for various types of observed data. These likelihood calculations permit inference of the strength and variability of transmission for individual cases, in terms of 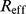 and 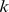. All calculations were conducted with either Matlab or R. Code for all analyses is available at: https://github.com/sbfnk/nbbpchainsizes.

### Determining model parsimony when comparing two sets of data

**Figure 1:**
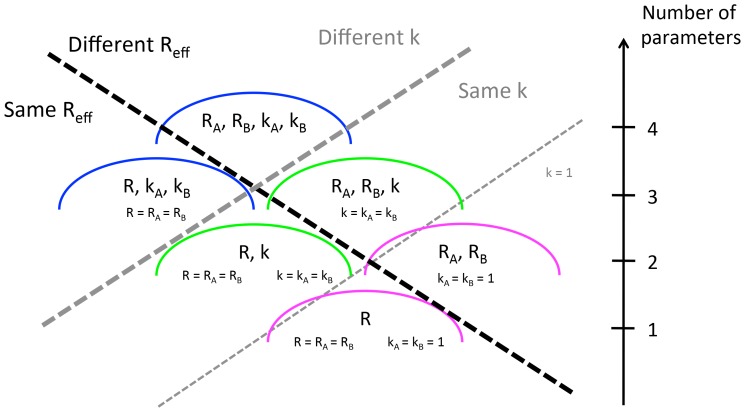
Six ways of modeling the transmission of two populations whose transmissibility is being compared. The dashed lines distinguish the models according the assumptions that are made about whether 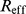 and the dispersion parameter are the same or different for the two populations. The axis on the right indicates the number of parameters used in each model. This is the sum of the number of parameters used to model 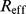 (either 1 or 2) and the number of parameters used to model dispersion (either 0, 1 or 2).

By calculating the likelihood of an observed set of transmission events, we can probe whether there is statistical support for differences in transmission between two pre-specified populations, 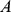 and 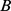. In our general model, the two types of individuals have distinct negative binomial characterizations and thus there are four parameters in total. We label these four parameters 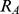, 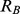, 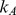 and 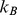 with the subscripts corresponding to the type of individual. Five simpler models that are nested within the 4-parameter model can be constructed by assuming 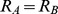, 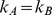 and/or 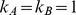 (Figure 1). The specific test case of 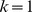 is chosen for the nested models because this corresponds to a geometric offspring distribution which is the expectation for a traditional SIR or SEIR model. These models assume homogenous mixing with constant infectivity over an exponentially distributed infectious period [29]. For each model, we determine the parameter values (MLE) that maximize the log-likelihood. The 95% confidence intervals and confidence regions shown in the figures were found by profiling on 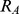 and/or 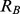 and employing the likelihood ratio test [30]. Model comparison is accomplished via the Akaike Information criterion (AIC) [31].

To identify whether there is statistical support for a difference in 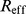 for two data sets, the AIC scores were computed for all six aforementioned models. A difference in 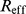 was deemed statistically significant according to the rule that the model with the best AIC score cannot be within two AIC units of a model that supports identical values of 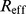 for the two sets of simulations. This rule is in approximate alignment with the commonly used likelihood ratio test for establishing statistical support for the use of an extra parameter with 95% confidence, but we could not employ the likelihood ratio test explicitly because some pairs of models we consider are not nested. We verified the internal consistency of our modeling framework by applying this method to simulated data (Supplementary material, Text S1).

We used parametric bootstrapping to evaluate the type I error and the power for detecting a change in 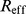 for our analyses. Specifically, for every analysis we simulated 20,000 new data sets. Each simulated data set replicated the two populations involved in the analyses (e.g. MERS-CoV chains before and after June 1, 2013). Two models were simulated. Half of the simulations used two distinct values of 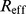 and 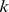 that matched the inferred values of our unrestricted four-parameter model. The other half of the simulations used a single value of 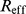 and 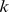 that matched the inferred values of our two-parameter model, which requires both 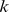 and 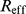 to be the same for all cases seen in the observed data. Our inferential algorithm for ascertaining a statistically significant difference in the inferred value of 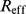 was then applied to all simulations. The type I error of an analysis (i.e. the probability that the analysis would falsely claim that 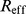 is different for the two types of cases considered) was estimated as the proportion of simulations based on the two-parameter model that were found to have a statistically significant difference in 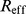 for the two types of cases. The parametric bootstrap probability (or power) of detecting a change in 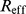 was estimated as the proportion of simulations based on the four-parameter model that were found to have significant difference in 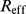 for the two types of cases.

## Results

Data used to generate all results can be found in the supplemental material (Text S2).

### The apparent trend towards decreased human-to-human transmission of MERS-CoV during the second half of 2013 may be a reflection of stochasticity rather than a true decrease in *R*_eff_

Since 2011, there have been over 500 confirmed cases of MERS-CoV, and over 140 associated deaths, suggesting a case fatality rate of 28% [32]. The persistent occurrence of small outbreaks is due to zoonotic spillover [33]–[35]. MERS-CoV may be a new virus, as the most recent common ancestor of viral samples from infected patients was estimated to have occurred after September 2010 [34]. The novelty of this virus and its high case fatality rate underscore the significance of monitoring the transmission of MERS-CoV. Although human-to-human transmission has been relatively limited so far, with 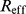 likely less than one, there is concern that future adaptation that could lead to spread similar to sudden acute respiratory syndrome (SARS) in 2003. Health authorities have prudently instituted a variety of infection control policies and procedures and a trend towards decreasing 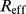 has been reported [34]. Since verification of the effectiveness of control has important implications, we reconsidered the evidence for a trend towards decreasing 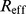.

**Figure 2:**
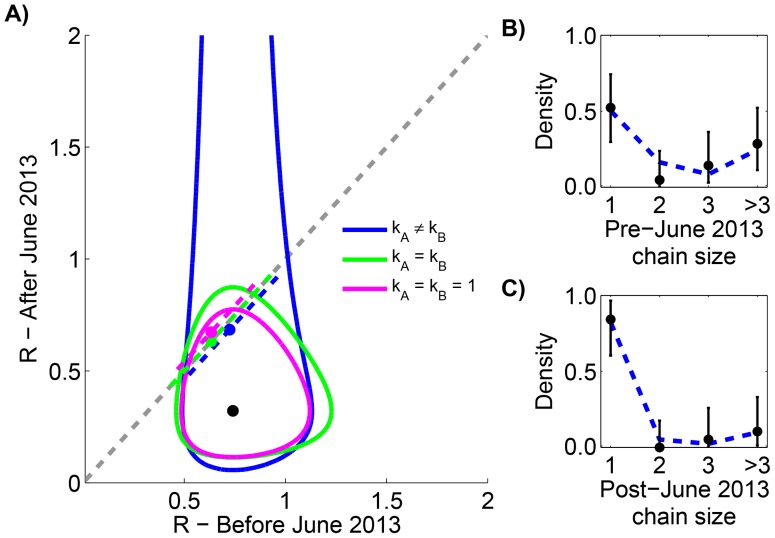
Assessing temporal variation of MERS-CoV transmission in the Arabian Peninsula before Aug 8, 2013. A) The results of estimating the effective reproduction number, 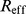, for six inter-related models of transmission are shown. The contours show the 95% confidence regions for three models that allow different values of 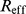 for cases occurring before versus after June 1, 2013. The distinction is that each model makes different assumptions about the degree of transmission heterogeneity (as explained in the text). The black dot shows the maximum likelihood estimation (MLE) estimate of the 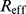 values for these three models. The dashed grey line indicates when 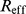 does not change with time. The dashed colored lines show the MLE estimate and 95% confidence interval of 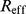 for the three models that assume transmissibility of cases is independent of time. The slight displacement of the colored lines from the dashed grey line is only for visual clarity. B) The fit of our preferred model to the early MERS-CoV chain size data is shown (Table 1). The error bars of the data correspond to 95% confidence intervals as determined by non-parametric bootstrapping of chain sizes. C) The fit of our preferred model to the late MERS-CoV chain size data is shown.

To avoid artifacts of assembling multiple data sources, we restricted our analysis to the previously reported chain size distribution for all MERS-CoV cases in the Arabian Peninsula occurring before August 8, 2013 [34]. Previous analysis of these data shows that 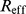 is 0.74 (95% CI 0.53–1.03) before June 1, 2013 and 0.32 (95% CI 0.14–0.65) after June 1, 2013. Our results replicate the finding that independent evaluation of cases before and after June 1, 2013 results in an estimate of 0.7 and 0.3 for 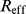 respectively (Figure 2 and Table 1). When our six models are compared, we do not find statistical support for models with different values of 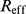 before and after June 1, 2013. This is again consistent with the results of prior studies that determined a p-value of 0.07 for change in 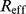, but our analysis allows the possibility of a high degree of transmission heterogeneity.

**Table 1:** Inference results for assessing temporal variation of MERS-CoV transmission in the Arabian Peninsula before Aug 8, 2013.

Restrictions	Parameters	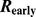	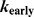	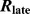	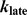	Log likelihood	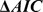
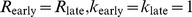	1	0.6	1	0.6	1	−59.6	2.3
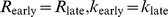	2	0.6	0.5	0.6	0.5	−59.4	3.8
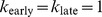	2	0.7	1	0.3	1	−57.5	0
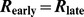	3	0.7	6.5	0.7	0.1	−57.2	1.4
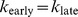	3	0.7	1.3	0.3	1.3	−57.5	2
None	4	0.7	6.8	0.3	0.2	−56.5	2.1

Maximum likelihood parameters, log-likelihood scores and AIC values are shown for the various inference methods described in the text. 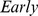 and 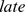 corresponds to transmission before and after June 1, 2013, respectively. The left column identifies the method by indicating the parameter constraints used. The model with 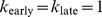 is chosen as the reference point for the 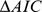 calculations since it has the best overall AIC score. Although this model has the best 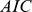 score, it assumes two different values of 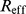. Erring on the side of requiring 95% confidence to distinguish 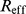 values, our method does not indicate that there is enough statistical support for using two different values of 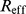 because the 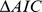 score of the 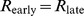 model is less than two. Thus the 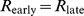 is our preferred model (indicated by the bold cell). There were a total of 81 early cases among 21 chains and a total of 28 late cases among 19 chains.

### *R*_eff_ is significantly different between transmission of measles in the United States (1997–1999) and Canada (1998–2001)

Local elimination of measles is dependent on vaccination programs, and the potential for re-emergence necessitates continued surveillance and re-assessment of vaccination strategy [1], [3], [36]–[38]. Even where elimination has been achieved, there can be sporadic clusters of infection due to a combination of geographic importation and pockets of susceptibility [39]–[41]. Geographical differences in transmission may arise due to differences in cultural practices, public health guidelines, population density and other factors. Methods that delineate whether differences in 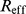 are statistically significant for two different regions can therefore help to identify key differences in transmission potential and thus pinpoint opportunities for improved control.

**Figure 3:**
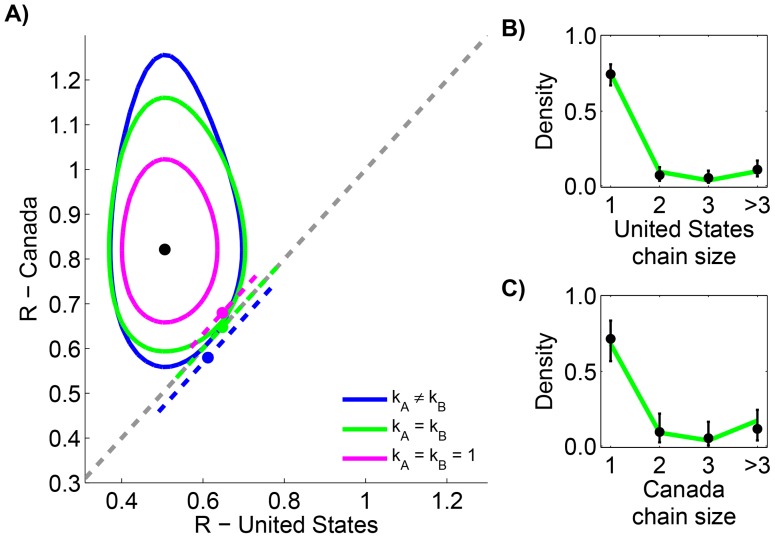
Comparing the transmissibility of measles in the United States (1997–1999) and Canada (1998–2001). The layout is analogous to Figure 2, except that the axes represent locations rather than time periods. Because of the ambiguity in the correct classification of an infection cluster with six cases that had two primary infections, this data point was not included in panels B and C (but it was included in the calculations for panel A, as our method accounts for all possible chains in this cluster).

Measles data in the United States (1997–1999) and Canada (1998–2001) are reported according to the size of infection clusters [39], [40]. Most infection clusters have a single primary infection, but even when multiple primary infections exist (as in the case of a cluster with six cases in the United States), the likelihood calculation needed for assessing differences in 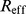 is straightforward (Supplementary Material, Text S1). When the two data sets are compared, the results indicate that 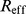 for the United States and Canada are significantly different (Figure 3 and Table 2). Meanwhile, the results also confirm previous studies that infer a high degree of transmission heterogeneity in measles transmission [19], [23]. This can be seen from Table 2 since the MLE estimates for 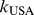 and 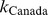 are less than one and the 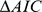 value of the model with 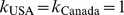 is large. On the other hand, there is negligible statistical support for distinct values of 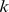 in the two countries. The type I error for this situation was estimated to be 4.9% by parametric bootstrapping.

**Table 2:** Inference results for comparing the transmissibility of measles in the United States (1997–1999) and Canada (1998–2001).

Restrictions	Parameters	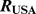	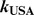	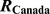	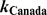	Log likelihood	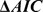
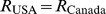							
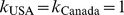	1	0.65	1	0.65	1	−270.7	21.9
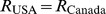							
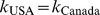	2	0.65	0.23	0.65	0.23	−261.0	4.5
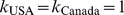	2	0.51	1	0.82	1	−263.7	10.0
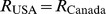	3	0.61	0.29	0.61	0.15	−260.5	5.6
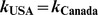	3	0.51	0.28	0.82	0.28	−257.7	0.0
None	4	0.51	0.32	0.82	0.21	−257.5	1.6

The layout is analogous to Table 1. Although the ‘None’ model, has a 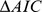 within two (i.e. our chosen threshold for statistical significance) of the best model, our analysis still suggests that 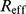 is statistically different for the United States and Canada, because both of these models have distinct 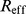 values for the two countries. Thus the 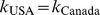 model (bold cell) is our preferred model. There were a total of 336 cases in the United States among 166 chains and a total of 274 cases in Canada among 49 chains.

### Significant differences existed between primary and secondary transmission of smallpox in Europe, 1958–1973

Smallpox is the only human disease to have been eradicated and thus represents a tremendously successful use of control [12]. During the endgame of smallpox eradication in the middle of the 20th century, smallpox cases in Europe resulted in rapid implementation of quarantine and control procedures. Transmission data for smallpox infections in Europe that occurred during this period provide an opportunity to investigate how control interventions impacted the transmissibility of primary cases caused by geographic importation relative to secondary cases resulting from local transmission [12].

**Figure 4:**
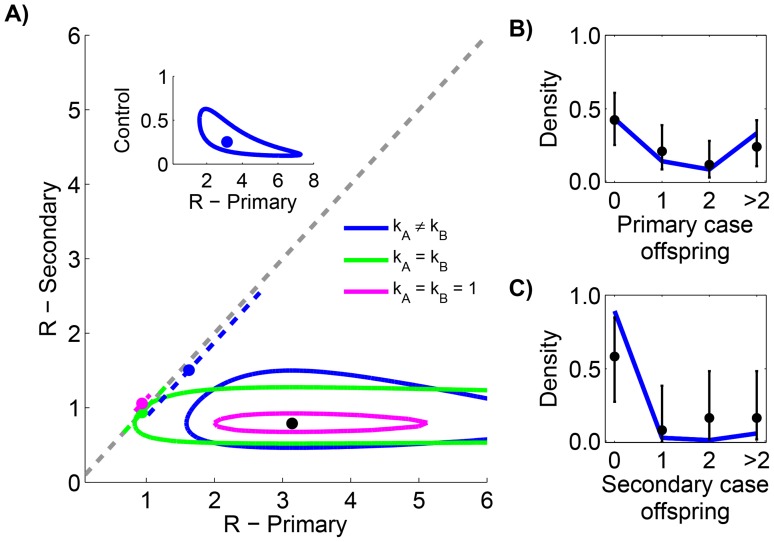
Comparing the transmissibility of primary and secondary cases for smallpox in Europe, 1958–1973. The layout is analogous to Figure 2 except the axes distinguish between transmission of primary and secondary cases. The inset of panel A replicates the results when 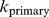 and 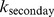 are inferred separately (our preferred model), except that the y-axis is now the ratio of 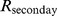 to 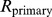. For panels B and C, the data is shown only for cases where there was a clear record of subsequent secondary infections (as opposed to knowing that four cases lead to ten secondary cases in aggregate). The 95% confidence intervals were found by parametric bootstrap on this more limited data set.

Smallpox clusters were tabulated according to the number of cases in each generation of spread [12]. The inference results indicate that secondary cases transmitted significantly less than primary cases (seen by the lack of overlap of contours with the grey line in Figure 4 and by the statistical selection of the non-restricted model in Table 3). In fact, the effectiveness of control procedures can be quantified by looking at the ratio of reproduction numbers for primary and secondary transmission (Figure 4 inset). The ratio of the maximum likelihood values for 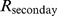 to 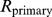 suggests that control reduced 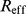 by 75%. Meanwhile, for both primary and secondary transmission, a high degree of transmission heterogeneity is evident (since the MLE estimates of 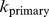 and 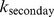 are substantially less than one and the 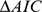 value of the 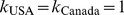 model is large). Based on selection of the unrestricted model, and the associated estimates of 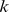, there appears to be significantly more heterogeneity of disease transmission for secondary cases than for primary cases. The type I error for this analysis was estimated to be 5.1% by parametric bootstrapping.

**Table 3:** Inference results for comparing the transmissibility of primary and secondary cases for smallpox in Europe, 1958–1973.

Restrictions	Parameters	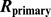	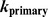	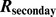	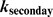	Log likelihood	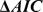
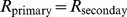							
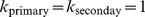	1	0.94	1	0.94	1	−427.8	415.4
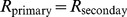							
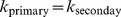	2	0.94	0.06	0.94	0.06	−236.5	34.8
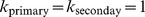	2	3.14	1	0.79	1	−400.8	363.5
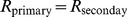	3	1.63	0.30	1.63	0.03	−223.6	10.9
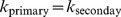	3	3.14	0.06	0.79	0.06	−233.7	31.2
**None**	4	3.14	0.37	0.79	0.04	−217.1	0.0

The layout is analogous to Table 1. There were a total of 36 primary cases and 537 secondary cases.

### Differences between primary and secondary transmission of human monkeypox in the Democratic Republic of Congo (1981–1984) appears negligible

Following the eradication of smallpox in 1979, the World Health Organization was concerned that subsequent cessation of smallpox vaccination would allow other diseases to flourish [42]. Monkeypox was of particular concern because exposure to smallpox or smallpox vaccination provided protection against monkeypox. Estimates of 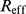, extrapolated from contact tracing data gathered during rigorous surveillance in the Democratic Republic of Congo (formerly Zaire) during 1981–1984, provided re-assurance that endemic transmission would not be sustainable even when population immunity to monkeypox waned [43].

The initial analysis of monkeypox transmission did not quantitatively compare the transmission of primary cases (i.e. those caused by animal-to-human transmission) to the transmission of secondary cases (i.e. those caused by human-to-human transmission). Since the characteristics of these cases differ (i.e. only primary cases required exposure to infected animals), differences in transmission are possible. Increased transmission of secondary cases could also arise from population structure [25], or evolutionary adaptation [8], [10]. For example, network models have proposed that social structure impacts the effective reproduction number of individual cases [44]–[48]. In particular, the random network model that we have considered (Supplementary material, Text S1) predicts that secondary cases transmit more than primary cases since highly-connected individuals are most likely to both acquire and spread infection. If this aspect of the random network model is accurate, the risk of endemic spread as population immunity wanes may be higher than previously expected. This is because 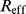 for secondary transmission would be expected to increase more than 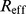 for primary transmission. It is thus important to ascertain whether there is a difference between primary and secondary transmission that is consistent with the random network hypothesis.

**Figure 5:**
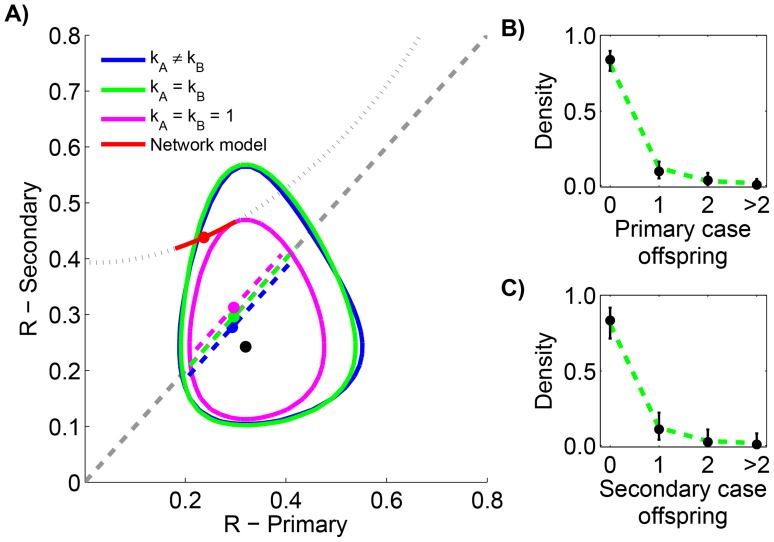
Comparing the transmissibility of primary and secondary cases for human monkeypox in the Democratic Republic of Congo, 1981–1984. The layout is analogous to Figure 4, except that the random network model has been added. The dotted line in panel A shows the relationship between 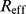 for primary and secondary infections in the random network model profiled on 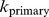. The red curve shows the 95% confidence interval for inference with the random network model. The data shown in panels B and C are limited to instances where the transmission links could be unambiguously counted.

As part of the monkeypox surveillance efforts, transmission was tabulated according to the number of cases in each generation of spread [43], [49]. These data can be used to ascertain whether there is a statistically significant difference in primary versus secondary transmission (Figure 5 and Table 4). The results indicate a lack of evidence for a difference between the 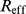 of primary and secondary cases (seen by noting the overlap of contours with the grey line in Figure 5 and because the preferred model in Table 4 has 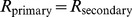). The low values for the maximum likelihood estimates of 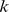 are consistent with previous studies that infer a high degree of transmission heterogeneity in monkeypox transmission [20], [23].

**Table 4:** Inference results for comparing the transmissibility of primary and secondary cases for human monkeypox in the Democratic Republic of Congo, 1981–1984.

Restrictions	Parameters	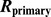	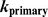	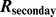	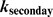	Log likelihood	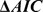
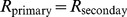							
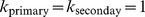	1	0.30	1	0.30	1	−137.8	4.3
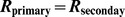							
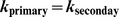	2	0.30	0.33	0.30	0.33	−134.7	0.0
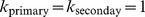	2	0.32	1	0.24	1	−137.5	5.6
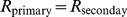	3	0.29	0.30	0.29	0.46	−134.6	1.8
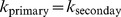	3	0.32	0.33	0.24	0.33	−134.4	1.5
None	4	0.32	0.30	0.24	0.72	−134.4	3.4
Random network	2	0.24	1.18	0.44	2.18	−143.0	16.5

The layout is analogous to Table 1, except that the random network model is added. There were a total of 147 primary cases and 62 secondary cases.

### The strength of animal-to-human transmission of monkeypox appears to be similar to human-to-human transmission

Animal-to-human transmission of monkeypox is an important contributor to overall disease burden. Determining the factors that allow continual introduction of monkeypox into human populations requires knowledge of how monkeypox maintains itself in reservoir hosts and the mechanisms that allow its transmission to humans [6], [50]. In this section we assess whether an infected animal in contact with humans has a distinct set of inferred transmission parameters than infected humans. The relationship between infection source and transmissibility is an active area of research for many multi-host diseases systems [51]–[55], particularly for zoonotic infections.

**Figure 6:**
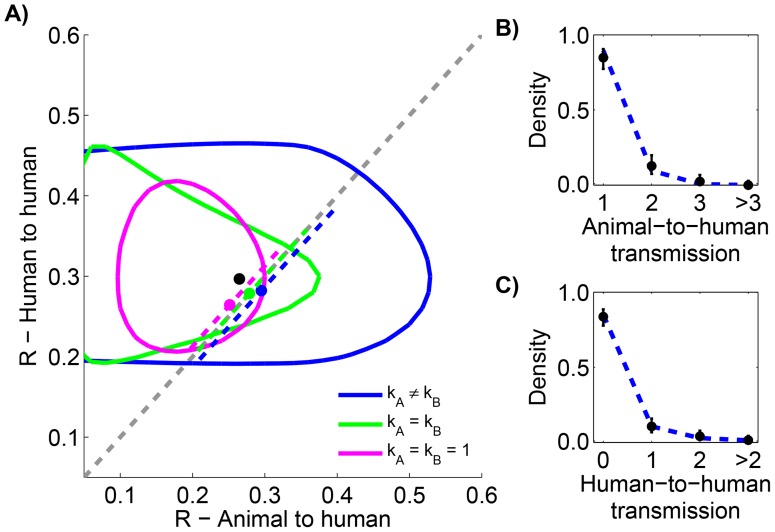
Comparing animal-to-human and human-to-human transmissibility for human monkeypox in the Democratic Republic of Congo, 1981–1984. The layout is analogous to Figure 5, but now the axes distinguish between animal and human transmission of monkeypox. The data shown in panel C is limited to instances where the transmission links could be unambiguously counted.

Since the infection cluster data for monkeypox contains information on how many primary infections are in each cluster, it can be used to infer the amount of animal-to-human transmission that occurs when infected animals make contact with humans. To accomplish this, we assume that the negative binomial offspring distribution that has been shown to be a good description of human-to-human transmission [23] is also an effective model of animal-to-human transmission. We let 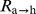 represent the average number of primary cases caused by an infected animal that has contact with humans. Our results indicate that the 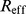 for human-to-human transmission is similar to 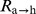 (Figure 6 and Table 5). There is also evidence that animal-to-human transmission is relatively homogeneous (since the 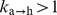 for the preferred model). If one takes the MLEs of 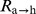 and 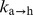 for the preferred model at face value, then we estimate that at least one infection occurs 25% of the time that a infected animal has contact with humans.

**Table 5:** Inference results for comparing animal-to-human and human-to-human transmissibility for human monkeypox in the Democratic Republic of Congo, 1981–1984.

Restrictions	Parameters	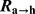	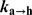	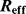	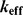	Log likelihood	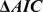
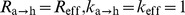	1	0.3	1	0.3	1	−175.6	1.9
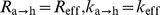	2	0.3	2.2	0.3	2.2	−174.9	2.4
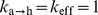	2	0.2	1	0.3	1	−173.7	0.0
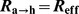	3	0.3	6.5	0.3	0.4	−172.8	0.1
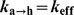	3	0.1	0.4	0.3	0.4	−172.9	0.5
None	4	0.3	3.4	0.3	0.4	−172.7	2.1

The layout is analogous to Table 1. The 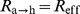 model was the preferred model since is within two of the model with the best 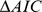 value, indicating there is not sufficient statistical support for distinct reproduction numbers. There were a total of 125 animal exposures leading to at least one primary case and 209 human cases. Despite the size of the data set, the 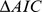 values are all quite small because there are large confidence intervals associated with the use of a truncated negative binomial distribution for inference [19].

### Quantifying the surveillance needs for detecting a difference in *R*_eff_ for monkeypox since the eradication of smallpox

Recently, a 20-fold increase in the incidence of monkeypox has been reported in the Democratic Republic of Congo [56], and there is concern that 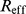 for monkeypox may have increased. The lack of cross-protective immunity to monkeypox from either smallpox vaccination or natural exposure to smallpox provides a mechanism for why 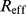 would increase [57]. However, land-use changes that impact the potential for animal-human transmission have also been suggested as a cause of an increase in monkeypox incidence [58], [59], and could do so without changing 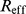. There are no active interventions in place for monkeypox, so it is important to determine if 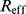 has changed in order to understand the source of increased incidence.

**Figure 7:**
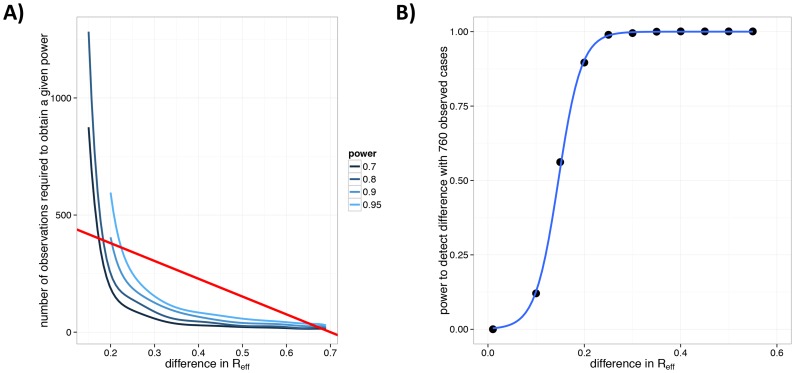
Power to detect a change in 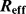 for human monkeypox following smallpox eradication. A) Number of observed chains of transmission for monkeypox needed to detect a change in 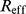 relative to 1980–1984. The 1980–1984 monkeypox data (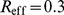, 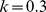) are compared against a set of simulations with 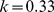 and 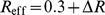, with 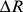 specified on the x-axis. This procedure was repeated 1000 times for each value of the number of simulated chains, 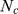 (as specified by the y-axis). For each value of 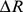, the blue lines indicate the lowest number of observations for which a given power (as a proportion of the 1000 simulations) was achieved. The shades of blue (see legend) indicate different levels of power for which this was done. The straight red line corresponds to the mean number of chains that would have been observed for the 760 case detected during the 2005–2007 monkeypox surveillance [56] for different values of 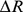. This line corresponds to 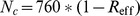 chains (since the average chain size is 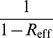 [20]). B) The power of the 2005–2007 monkeypox surveillance data to detect a change in 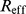 for monkeypox. The black dots are the results of simulations, the blue line is a smooth fit to these. This panel corresponds to a cross-section of the figure in panel A along the red line.

Due to logistical barriers and the rare nature of the disease, acquiring data on monkeypox is a challenge [42], [56]. In the wake of smallpox eradication, the infrastructure for monkeypox surveillance in 1980–1984 was strong and well funded [42]. The detailed transmission data from this surveillance effort provide an estimate of 0.30 for 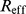 (95% CI: 0.21–0.42) and 0.33 for 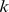 (95% CI: 0.17–0.75) [20]. For the 2005–2007 surveillance effort, specific data on cluster sizes and individual-level transmission are unavailable, so an assessment of 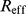 cannot be made. However, we can quantify the amount of data that would be needed in order to detect a change in 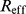 relative to 1980–1984 [42], [43], [49]. Simulations show that 200 clusters would provide 70% power to detect an increase in 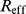 from 0.3 to 0.5 (Figure 7A). As the number of observations increase, smaller changes are more readily noticeable.

Consideration of the relationship between 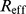, the number of chains and the number of cases provides perspective on the power of the recent surveillance efforts (2005–2007) to detect a change in 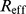 [56]. It appears that there is 95% power to detect an increase in 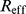 from 0.3 to 0.55 with analysis of the 760 observed cases (Figure 7B).

## Discussion

In summary, we have introduced and validated a method for comparing case data grouped into different categories and applied this method to a number of different scenarios. The versatility of the method has been explored through examination of a variety of diseases and data types. By providing quantitative information on transmission, surveillance needs, or the effectiveness of control interventions, each type of analysis has the potential to assist in epidemiological assessments and public health planning.

### MERS-CoV transmission

To reduce the burden of MERS-CoV and reduce the risk of global spread, effective control procedures are of obvious importance. Given the large amount of resources and effort that have already been directed towards the control of MERS-CoV, it would be reassuring to see a statistically significant decrease in 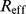. When analyzing data on MERS-CoV cases that presented before Aug 8, 2013, the unrestricted model had the best 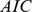 score. This unrestricted model suggested that because 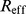 decreased from 0.7 to 0.3, control is over 50% effective. However, there is not enough data to show statistical significance for this result. Meanwhile, our analysis is likely biased by the large outbreak that initiated the observational period for the data, so further studies are needed to more accurately evaluate the impact of control interventions [60].

Unfortunately, the number of recent confirmed MERS-CoV cases remains significant and the overall incidence may be increasing [32]. An increase in the number of cases can be caused by an increased 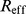, an increased rate of primary cases, or a combination of these effects [61]. Based on our observation that 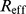 is more likely to be decreasing after June 2013 than increasing, the paradigm of emergence that is most consistent with the previously published data we have analyzed is that MERS-CoV incidence may be increasing in its non-human reservoir, but that human-to-human transmission remains stable. In fact, sequence data support the possibility of an expanding epidemic in animal hosts of MERS-CoV that could lead to an increased incidence of primary cases [34]. However, other factors, such as seasonal drivers of transmission could also impact the temporal trend of 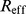. An increased case load could also be observed if transmission patterns have not changed much, but greater interest in and knowledge of MERS-CoV has led to improved surveillance. This could paradoxically lead to both an increase in the number of observed cases and a decrease in the observed value of 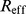 because of a greater chance of seeing a larger proportion of smaller outbreaks [19], [62].

Given the relative paucity of cases and uncertainties regarding case observation probability, it would be inappropriate to make a definitive statement concerning the cause of the apparent increase in MERS-CoV incidence at this time. However, as more data on MERS-CoV are reported, the types of analyses presented in this manuscript can be rapidly applied to address hypothesis-driven questions concerning the temporal trends of incidence and the impact of control intervention. In particular there may be concerns that certain subgroups of MERS-CoV cases may have increased transmission, such as those occurring in health care settings where nosocomial transmission is higher or in geographic regions where control interventions are harder to implement. Alternatively, as we have shown with smallpox, there may be a difference in the transmissibility of primary cases versus secondary cases. With more data, our method can help to quantify differences in transmission, and evaluate whether certain population subgroups may have an 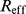 that exceeds the critical value of one. While it is not necessary for future data to be resolved to the level of individual transmission events, the types of analyses we have presented do require knowledge of chain size distributions rather than aggregate epidemic curve data. Meanwhile, an important gap in the currently available data is a quantitative assessment of the case reporting probability for MERS-CoV cases and whether this is increasing with time. Improved knowledge of the reporting probability would permit adjustments to the likelihood calculations and reduce the bias of imperfect case ascertainment [19].

### Measles transmission

Our comparison of measles transmission in the United States and Canada provides a framework for elucidating geographic differences in transmission (Figure 3). Interestingly, while our analysis supported a difference in 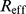 between the two countries, a difference in the degree of transmission heterogeneity (as quantified by the dispersion parameter) was not identified. This apparent disassociation between the strength of transmissibility and the mechanisms of transmission heterogeneity may occur if the heterogeneity is due to intrinsic biological processes such as variability in viral shedding. However, the relationship between the value of dispersion parameter and various mechanisms of transmission heterogeneity is not straightforward so the interpretation of similar values of dispersion is unclear.

There are many reasons why the value of 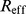 may differ between the United States and Canada. One consideration is a potential difference in the timing of the introduction of two-dose vaccination. The Advisory Committee on Immunization Practices and the American Academy of Pediatrics recommended two-dose coverage in 1989 [63]. Although the coverage in 2004 appeared similar between the United States and Canada [38], it is unclear whether this level of coverage was achieved at the same time in both countries. To assess whether a difference in vaccine coverage explains the difference in observed 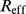 here, it would be helpful to run a similar analysis on more recent data. Other factors that could contribute to the difference in 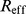 include a greater tendency in the United States to conduct contact tracing for susceptible cases and vaccinate close contacts, a greater sensitivity in Canada for reporting milder cases of measles, or greater difficulty of detecting isolated cases via passive surveillance in Canada [37], [38]. More detailed information of the impact of contact investigation, stratification of cases based on disease severity, and quantitative comparison of case ascertainment in passive versus active surveillance would provide additional insight.

### Smallpox transmission

Smallpox control is already known to have been very effective; however, our analysis of smallpox transmission in Europe around the time of eradication quantifies the impact of interventions for control (Figure 4) showing that there was a reduction of 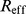 for secondary cases by 75% compared to primary cases. This effect of control may be an underestimate because it does not account for the possibility of late arrival of imported cases during the course of infection. Since the infectious period of imported primary cases may have occurred outside of the country of residence, the actual 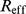 for primary cases might be higher than seen in the data and thus the effect of control may be even greater than our estimates indicate.

Here we have shown how 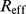 for each generation can be quantitatively compared, using published transmission data. Our analysis of differences in the transmissibility of cases as an outbreak develops is not unique (see for example [64]). However, previously published methods rely on symptom-onset data to determine 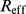 at various stages of an outbreak and thus these approaches could not be performed on the smallpox data set.

Aside from the change in 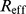, the marked increase in degree of transmission heterogeneity for secondary cases (as evidenced by a decreased in the observed value of 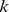) suggests that control tended to be individual-specific rather than population-wide. Here, individual-specific control refers to an intervention that is completely effective for 75% of cases but not effective at all for the remaining cases, whereas population-wide control refers to an intervention that reduces the transmissibility of each case by 75% [23]. For individual-specific control, a large number of cases become dead ends for infection so the observed degree of heterogeneity increases [19], [20]. In contrast, the observed degree of transmission (as quantified by the dispersion parameter) would not change for population-wide intervention. The support for individual-specific control is highly consistent with the quarantine and ring vaccination methods employed during smallpox elimination efforts [12]. These observations show how understanding the variation in both the strength and heterogeneity of transmission can provide insight into disease dynamics.

### Monkeypox transmission

Our analysis of monkeypox in the Democratic Republic of Congo demonstrates how our method can be used to inform surveillance planning. In particular, by determining the number of chains that needed to be observed in order to detect various degrees of change in 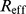, we provide perspective regarding the extent to which the 760 monkeypox cases observed between 2005 and 2007 [56] can provide enough information to detect increased transmissibility (Figure 7). Based on our power analysis, it appears that a change in 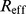 due to declining population immunity should be detectable, since 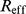 is expected to approach 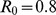 [43]. However, this result needs to be interpreted in context because our model assumes that the probability of case observation is high and that distinct infection clusters can be determined. Given the logistical challenges of recent surveillance efforts [56], these assumptions are unlikely to have been met, so the realized power for detecting a change in 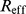 is probably lower. Nevertheless, this simulation analysis provides perspective concerning the trade-offs of thoroughness in detecting and characterizing cases versus observing cases within a greater catchment area for any future surveillance efforts for which measurement of 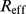 is of interest.

When we focused on more detailed generation-level data for monkeypox transmission from 1980–1984, we found no support for enhancement of 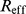 by highly-connected individuals in secondary generations (Figure 5). This suggests that the high degree of transmission heterogeneity may be caused by biological factors, rather than variability in social contact. However, a key assumption of the network model we tested is that primary cases are infected at random relative to their degree (as might reasonably be expected for a zoonotic infection). It may be that high-connected individuals are also more likely to get a primary infection. If this were the case, then highly connected individuals would contribute to heterogeneity of both primary and secondary transmission. Meanwhile, the lack of increased 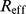 for secondary transmission provides assurance that significant viral adaptation is not occurring, although local depletion of susceptible individuals within small sub-networks such as households could obscure signals of viral adaptation.

We found that humans and animals in contact with humans produce similar numbers of human cases (Figure 6). Moreover, we estimated that 25% of human exposure to an infected animal lead to at least one detected human case. While the truncated negative binomial distribution produces unbiased estimates of transmission parameters, the confidence intervals can be quite large [19]. Furthermore, the *a priori* specification that the offspring distribution will be characterized by negative binomial distribution is a strong assumption. Thus the inferred proportion of animal-to-human exposures leading to infection deserves cautious interpretation. Nevertheless, this type of analysis could be useful for informing surveillance and detection efforts in wildlife species. In particular, since the overall incidence of monkeypox is quite low (14.42 per 10,000 per year [56]), the observation that there may be only 4-fold more infected animals in contact with humans than the number of observed infection clusters provides perspective on the fact that monkeypox virus has only been isolated from one wild animal (as of 2011) [58]. If contacts with infected animals account for a small proportion of overall human contact with reservoir species, the use of targeted-surveillance strategies that can exploit spatial-temporal data to identify likely hotspots of incidence [58], [59], [65] may be essential to improve detection efforts in wildlife hosts.

### Sensitivity to a small number of large transmission events

As with any model selection or measurement scheme, a small portion of the data, or even a single data point, can have a particularly large influence. For example, the largest transmission chain in the Canadian measles data consists of 155 cases while the second largest chain has just 30 cases. Moreover, the chain with 155 cases was associated with a religious community that resisted immunization, thus it could be argued that this chain is not representative of the population as a whole. If the 155-case chain were excluded from the 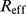 analysis, our method would no longer find statistical support for a difference in 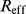 between the United States and Canada (Supplementary material, Text S1).

However, rather than excluding a possible outlier, our preference is to treat the data at face value. From a modeling perspective, it is often unclear whether the mechanism responsible for a purported outlier is absent in the rest of the data. For example, in the case of Canadian measles data set, the second largest chain of 30 cases was also associated with a religious community. In addition, a particularly large chain does not represent a single large transmission event, but rather an entire group of individuals who collectively had relatively high transmission. Mathematically, a high degree of transmission heterogeneity (represented by low values of 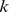) is expected to have a big tail for the distribution of the number of cases that each case causes [23]; thus, a large transmission event or chain in a set of data will increase the estimated value of 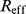, but will also decrease the estimated value of 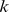. A lower 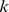 will be associated with a wider confidence interval for 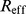 and this would make it harder for our analysis to find a statistically significant difference in 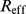 [19], [20]. Thus our modeling framework has a built-in mechanism that compensates for large transmission events and chains that are consequences of intrinsic population-level or individual-level mechanisms of heterogeneity.

### Impact of imperfect case observation

A key caveat of our analyses is that we have assumed perfect observation of cases. Some surveillance programs, such as measles in the United States, have documented evidence of high case observation [36]. However, this level of case ascertainment cannot be expected of all diseases, particularly those such as MERS-CoV that are quite new. Meanwhile, even meticulously collected data are prone to multiple sources of observation bias due to limited surveillance resources, subclinical infections, laboratory error, or other factors.

When the limitations of observation can be quantified, likelihood calculation for observed transmission events can be adjusted appropriately [19], [20]. The challenge is that the limitations of surveillance systems and case ascertainment are often difficult to quantify. An alternative to explicit correction of observation bias is to simply consider what level of observation bias would impact key results. For example, in our analysis of the difference between animal-to-human and human-to-human transmission of monkeypox, it is quite possible that a number of animal-to-human infections are unobserved — particularly if the resulting primary infection is mild and has no further transmission. When we treated observation of an infection cluster as an all-or-none process with an independent probability, 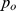, that each case would activate surveillance (thus implying many isolated cases would be unobserved), our preferred model of transmission remained stable even for a 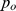 of 0.1 (Supplementary material, Text S1). This provides re-assurance that our methodology is not necessarily sensitive to imperfect observation. However, different data sets or a different type of observation bias could yield less stable results.

### Other modeling extensions

In our analyses, we have allowed for at most two values of 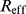 and 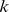 in a data set rather than permitting additional stratification or a continuous distribution of values. These simplifications are not always valid assumptions. However, modifications to the likelihood calculation can often be made in order to accommodate more complicated data sets so that our framework for detecting a difference in 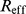 can be utilized. For example, the offspring generating function used for the likelihood calculation can be written in terms of a continuous variable that provides a smooth transition between the extreme limits of classification. In fact this approach has been used to investigate whether there is a temporal trend of measles transmissibility in the United States [61].

Although we have mainly focused on differences in 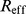 between two populations, our method can also be used to identify whether these populations differ in the observed degree of heterogeneity. Clustering of individuals with higher transmissibility may favor models with two distinct values for 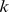 whenever two distinct values of 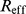 are observed. Meanwhile, situations that would favor a model with two distinct values of 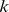 and one value of 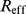 could arise if different mechanisms of control were used to maintain 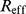 below a given threshold, as seen in the smallpox example. Regardless of which model is the preferred model for a given data set, the estimated or assigned value of 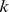 can be useful to assess the overall degree of transmission heterogeneity and the likely presence of super-spreaders [20], [23]. On the other hand, the specific mechanism of heterogeneity (e.g. differences in transmission potential among cases versus clustering of susceptible individuals) cannot be ascertained from estimation of 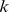 alone.

### Limitations

Our analysis is focused on determining whether there is statistical support for a difference in 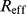 for individuals having a specific trait. Also, as exemplified by our direct comparison to the random network model (Figure 5 and Table 4), we can evaluate specific models of transmission. However, in the absence of a mechanistically derived model, our analysis cannot identify the cause of differences in 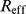. For-example, population-level factors favoring transmission (e.g. increased human density) cannot be directly distinguished from biological factors (e.g. evolutionary adaptation). Furthermore, the decrease in secondary transmission due to local depletion of susceptibles cannot be directly distinguished from decreases due to control mechanisms. Instead, our method needs to be considered as a tool that can identify differences in transmission (e.g. temporal trends for MERS-CoV, and geographic distinctions in measles) or quantify changes in transmission that are expected to occur (e.g. decreased transmission due to quarantine of smallpox cases or ring vaccination).

### Conclusions

By addressing diverse questions within varied data sets, we have demonstrated that a set of inter-related models within a branching process framework allows rigorous statistical assessment of whether particular characteristics of infectious cases impact transmission potential. We have focused on subcritical diseases, in large part because the type of surveillance data gathered for these diseases is most compatible with our computational approach. For MERS-CoV, we evaluated the possibility of a temporal trend towards decreasing 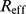 that may indicate stronger control, but did not find enough statistical evidence to confirm this finding. For measles, we found evidence of geographic variability that provides potential insight into the effectiveness of surveillance and public health interventions. For smallpox, we identified signatures of effective control by comparing primary and secondary transmission. For monkeypox, we found that the most parsimonious models are ones that incorporate a high degree of transmission heterogeneity, but do not differentiate between animal-to-human transmission, transmission of primary cases, and transmission of secondary cases. In general, the statistical support we observed for models that allow flexible inference of both 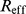 and 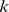 reinforces the importance of quantifying both the strength and variability of disease transmissibility. By providing a diverse array of applications and analyses, the method we have demonstrated can increase the value of existing surveillance data and improve strategies for future data collection. Through identifying specific risk factors for transmissibility and by assessing different sources of transmission heterogeneity, we hope that disease monitoring and control interventions can become more targeted and thus more effective.

## Supporting Information

Text S1 **Methods supplement.** Contains 1) A detailed description of the likelihood calculations 2) Simulation-based validation of the method 3) Sensitivity of measles analyses to a single large chain and 4) An analysis of imperfect observation of monkeypox transmission.
(PDF)

Text S2 **Data supplement.** Provides tables and references for all the data used in the analyses.
(PDF)
