# Supplementary material for: Detecting Differential Transmissibilities That Affect the Size of Self-Limited Outbreaks
Source: PLoS Pathog. 2014 Oct 30;10(10):e1004452. doi: 10.1371/journal.ppat.1004452 (PMC4214794; doi:10.1371/journal.ppat.1004452)
Supplement: Text S1 — Methods supplement. Contains 1) A detailed description of the likelihood calculations 2) Simulation-based validation of the method 3) Sensitivity of measles analyses to a single large chain and 4) An analysis of imperfect observation of monkeypox transmission. (PDF) [file ppat.1004452.s001.pdf]

Supplementary material: Detecting differential transmissibilities that affect the size of self-limited outbreaks

Seth Blumberg, Sebastian Funk, Juliet R. C. Pulliam

August 31, 2014

1 Likelihood calculations

To allow estimation of R_{eff} and k, the infected cases in a given population must be divided into independent subunits, such as generations of cases, transmission chains or infection clusters. We define a primary case as a case whose infection is external to the population of interest (e.g. due to importation from a foreign country or transmission from an infected animal). We define the first generation of cases to be all those infected by primary (i.e. zero-generation) cases, the second generation of cases to be all those infected by first generation cases, and so on. We define a chain to be a single primary infection plus all subsequent infections that are linked to it. We define an infection cluster as a group of chains that overlap in time and space so that the constituent chains may be hard to discern (sometimes referred to as an outbreak in the literature). The subunit definitions used in the analysis for each of the data sets considered in this manuscript are as follows:

- For the analysis of smallpox (Section 3.3 of the main text) and human-to-human transmission of monkeypox (Section 3.4 of the main text), the data consist of descriptions of infection clusters in which the number of cases in each generation of transmission is provided. Thus each transmission subunit consists of one generation of spread in a cluster and can be described as i individuals infecting j other individuals. The corresponding likelihood calculation is given in Section 1.1.

- For the analysis of MERS-CoV transmission in the Arabian Peninsula (Section 3.1 of the main text) as well as measles transmission in the United States and Canada (Section 3.2 of the main text), the only available data are the number of cases epidemiologically linked within a single transmission chain. Thus each transmission subunit consists of a complete transmission chain and can be described by the number of cases, j, in the chain. The corresponding likelihood calculation is given in Section 1.2. This likelihood is also used for the simulation studies of Section 2 and Section 3.6 of the main text.

- For the analysis of animal-to-human transmission of monkeypox (Section 3.5 of the main text), transmission subunits consist of the observed number of primary infections, m, in a cluster. It is assumed that all primary infections came from a single exposure (or 'point source') event.

Since the exposure events are recorded only when they lead to at least one primary infection, adjustments have to be made for all the unknown exposure events that resulted in no primary infections. The corresponding likelihood calculation is given in Section 1.3. For the human-to-human transmission that these data are compared to, the subunits are infection clusters with m primary cases and j cases in total (Section 1.2).

For the examples we consider in this manuscript, all transmitting individuals within a single transmission subunit have the same values of the transmission parameters, R_{eff} and k. However, the cases that are infected by the individuals in a transmission subunit may have a different set of transmission parameters. As a special case, we show how the transmission parameters of linked transmission units inter-relate for a random network model of social mixing (Section 1.4) and apply it to the generation-by-generation data of monkeypox (Section 3.4 of the main text).

The probabilities for each subunit in an observed set of transmission events are multiplied together to obtain the overall likelihood of the model given the data, and the likelihood is maximized over the model parameters.

1.1 Likelihood of a single generation of transmission

A classic result of branching process theory is that the coefficients of $Q(s)^i$ provide the probabilities that i cases collectively generate $0, 1, 2, \dots$ cases [1, 2]. This occurs because multiplication of two generating functions produces all possible pairs of terms, which is analogous to considering all possible ways that two cases can generate a given number of new cases. By exploiting the helpful property that differentiating $Q(s)^i$ shifts the coefficients leftwards and that evaluating the resulting function at $s = 0$ provides the value of the constant term, we can extract the likelihood (equivalent to the probability of the data given the model), $l_{i \to j}$, that i cases produce j infections,

$$l_{i \to j}(R_{\text{eff}}, k) = \frac{1}{j!} \left. \frac{d^j Q(s)^i}{ds^j} \right|_{s=0} \tag{1}$$

The generating function of a negative binomial distribution can be algebraically summarized in a closed form equation,

$$Q(s) = \left(1 + \frac{R_{\text{eff}}}{k}(1 - s)\right)^{-k}. \tag{2}$$

Applying equation 1 to our specific use of a negative binomial distribution for the generating function gives

$$\begin{aligned} l_{i \to j}(R_{\text{eff}}, k) &= \frac{1}{\Gamma(j+1)} \frac{d^j}{ds^j} \left(1 + \frac{R_{\text{eff}}}{k}(1 - s)\right)^{-k \cdot i} |_{s=0} && (3) \\ &= \frac{\Gamma(j + ki)}{\Gamma(j+1)\Gamma(ki)} \left(\frac{k}{R_{\text{eff}} + k}\right)^{ki} \left(\frac{R_{\text{eff}}}{R_{\text{eff}} + k}\right)^{j}. && (4) \end{aligned}$$

where Γ denotes the gamma function and thus $\Gamma(x + 1) = x!$. This is equivalent to the probability mass at j of a negative binomial distribution with a mean of $(R_{\text{eff}} i)$ and a dispersion parameter of (ki). This property can be understood intuitively. If we focus on cases where k is an integer, this relates to the interpretation of the negative binomial distribution as the number of tails that

occur in a sequence of coin flips before k heads occur. In our case the coin has a probability of $\frac{k}{R_{\text{eff}}+k}$ for flipping as a head. Repeating this process with i coins (analogous to observing how many offspring are generated by i cases), is analogous to flipping one coin until ki tails occur, which is just a new negative binomial distribution with the aforementioned scaling of the mean and dispersion parameter.

1.2 Likelihood of an infection chain or infection cluster

We use the superscript C to denote that we are now considering the likelihood of a complete *total* chain (or cluster), rather than the number of infections caused by a specified number of cases. When all cases of a chain have the same R_{eff} and k, the likelihood, l_j^C for a chain of size j is determined by noting that a single primary case leading to j infections amounts to j cases causing $j-1$ infections. This is because all cases except the first must be caused by one of the j total cases. This needs to be corrected by a factor of $\frac{1}{j}$ to account for the observation that only certain combinations of transmission events will yield chains that go extinct when there are exactly j cases [3].

$$l^C_{1\to j}(R_{\text{eff}},k) = \frac{1}{j} l_{j\to j-1}(R_{\text{eff}},k). \tag{5}$$

When transmission chains get entangled together into clusters (i.e., cases overlap in space and time), it is often the case that only the number of primary infections, m, and the total size, j, of the cluster are known. In this case the likelihood is found by noting that j cases cause $j-m$ infections. Meanwhile, the normalization factor for the requirement of proper extinction is $\frac{m}{j}$ [4]. Then,

$$l^C_{m\to j}(R_{\text{eff}},k) = \frac{m}{j} l_{j\to(j-m)}(R_{\text{eff}},k). \tag{6}$$

1.3 Likelihood of observing primary infections from a point-source exposure

A point-source exposure for primary infection occurs when there is a single event at which multiple individuals are infected from a single source. For example, multiple people could be infected with a zoonotic infection through butchering and/or consumption of a single infected animal. In analogy to what has been done for human-to-human transmission [5], we model the number of human infections caused by an infected animal that makes contact with humans as coming from a negative binomial distribution with mean $R_{\text{a}\to\text{h}}$ and dispersion parameter $k_{\text{a}\to\text{h}}$. However, since animal-to-human exposures are only observed when at least one human infection occurs, the probability of observing j cases from a point-exposure event is determined by normalizing the true probability of j transmission events by the probability that no animal-to-human transmission occurs. The probability that no transmission occurs is $l_{1\to 0}(R_{\text{a}\to\text{h}}, k_{\text{a}\to\text{h}})$. Thus the likelihood of observing j cases resulting from a point-source exposure (denoted with the superscript P) is,

$$l^P_j(R_{\text{a}\to\text{h}}, k_{\text{a}\to\text{h}}) = \frac{l_{1\to j}(R_{\text{a}\to\text{h}}, k_{\text{a}\to\text{h}})}{1 - l_{1\to 0}(R_{\text{a}\to\text{h}}, k_{\text{a}\to\text{h}})}. \tag{7}$$

Given specific values of $R_{\text{a}\to\text{h}}$ and $k_{\text{a}\to\text{h}}$, the probability that an animal-human exposure leads to at least one primary cases is $1 - l_{1\to 0}(R_{\text{a}\to\text{h}}, k_{\text{a}\to\text{h}})$.

1.4 Likelihood of a random network model

Our random network model tests a specific prediction about how transmission changes between primary and secondary cases. The model assumes that primary cases are infected at random and that secondary infection occurs in proportion to the number of contacts an infected individual has [6]. Thus there is an implicit assumption that heterogeneity in disease transmission is entirely due to variability in the number of social contacts. This implies that secondary cases may transmit more than primary cases because the individuals with the most contacts are most likely to become secondary cases and in turn spread disease to many others. For consistency with the prior likelihood calculations, we assume a negative binomial distribution for the number of social contacts each individual has. Accordingly, the generating function, $F_c(s)$, for the number of social contacts is,

$$F_c(s) = \left(1 + \frac{\mu}{k'}(1-s)\right)^{-k'}$$

where μ is the mean number of contacts and k' is the dispersion parameter for the contact distribution. We let T denote the constant probability of infection per contact. The number of cases generated by a primary case is found by binomial sampling over the number of contacts. Binomial sampling can be represented via the generating function as $G_p(s) = F_c(1 - T + Ts)$ [7]; therefore the generating function for the transmission of primary cases is

$$G_p(s) = \left(1 + \frac{\mu T}{k'}(1-s)\right)^{-k'}$$

This is equivalent to a branching process model in which $R_{\text{eff}} = \mu T$ and $k = k'$.

The distribution function for the number of contacts that an infected secondary case has, $F_s(s)$, is different from the one for primary cases for two reasons. First, each case loses one susceptible contact because they were infected by someone. Second, to account for the linear correlation between risk of infection (which we assume to be proportional to the number of contacts) and the number of subsequent individuals one case will be able infect, the distribution needs to be weighted accordingly. With these adjustments, the properly normalized generation function is [7,8],

$$F_s(s) = \frac{1}{\mu} \cdot \frac{dF_c(s)}{ds} = \left(1 + \frac{\mu}{k'}(1-s)\right)^{-k'-1}$$

As above, the generating function for the number of infectious offspring of secondary cases is obtained by binomial sampling of contacts. Accordingly,

$$G_s(s) = F_s(1 - T + Ts) = \left(1 + \frac{\mu T\left(1 + \frac{1}{k'}\right)}{k'+1}(1-s)\right)^{-k'-1}$$

$G_s(s)$ is equivalent to a negative binomial distribution with

$$R_{\text{eff}} = \mu T \cdot \left(1 + \frac{1}{k'}\right) \tag{8}$$

$$k = k' + 1. \tag{9}$$

This provides the mathematical formalism showing that R_{eff} is greater for secondary cases than for primary cases in the random network model. The difference in primary and secondary transmission is more pronounced for small values of k.

2 Simulation-based validation of the method

Simulations of transmission chains based on a known negative binomial offspring distribution were used to verify our ability to detect changes in R_{eff} (Figure S1). Each simulation produced a distribution of chain sizes for a pair of R_{eff} and k values. Pairs of simulations having different values of R_{eff} were identified according the methods in Section 2.2 of the main text. As data become more plentiful, the power to detect smaller differences in R_{eff} increases. The power decreases slightly as k decreases from 1 to 0.25 because a higher degree of transmission heterogeneity (i.e. lower k) results in wider confidence intervals for the inferred value of R_{eff} [15].

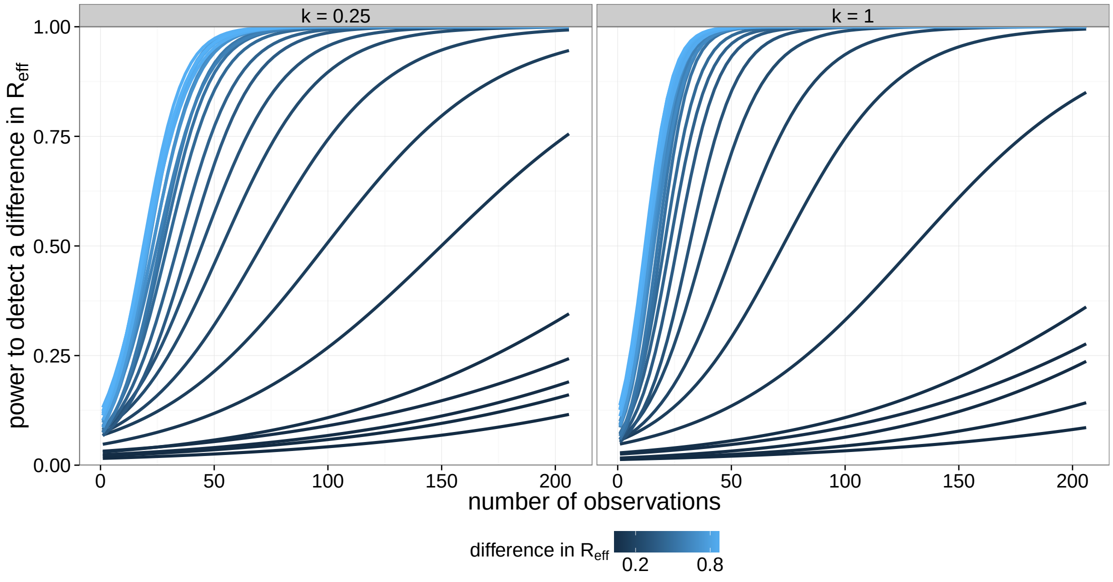

Figure S1: **Power of detecting a difference in R_{eff}.** Results from comparing two sets of simulations of chain size distributions that differ in the true value of R_{eff}. The number of observed chains in each simulation varied as indicated by the x-axis. A baseline simulation always had $R_{\text{eff}} = 0.1$ and $k = 0.25$ (left panel) or $k = 1$ (right panel). The comparison simulation had an R_{eff} that was greater than the baseline by the amount indicated by the color bar. The y-axis denotes the power to detect a difference in R_{eff}, or the proportion of simulation pairs for which a significant difference in R_{eff} was detected (i.e. the best-fitting model had different R_{eff}). The curves have been smoothed to improve legibility.

To ensure that our statistic for detecting a difference in R_{eff} (as described in the methods section of the main text) was not overly sensitive (resulting in a high probability of Type I errors), we also compared simulations which had identical R_{eff} (Figure S2). Consistent with our expectations, comparisons of simulations having the same R_{eff} show that we failed to reject the null hypothesis

with 95% confidence.

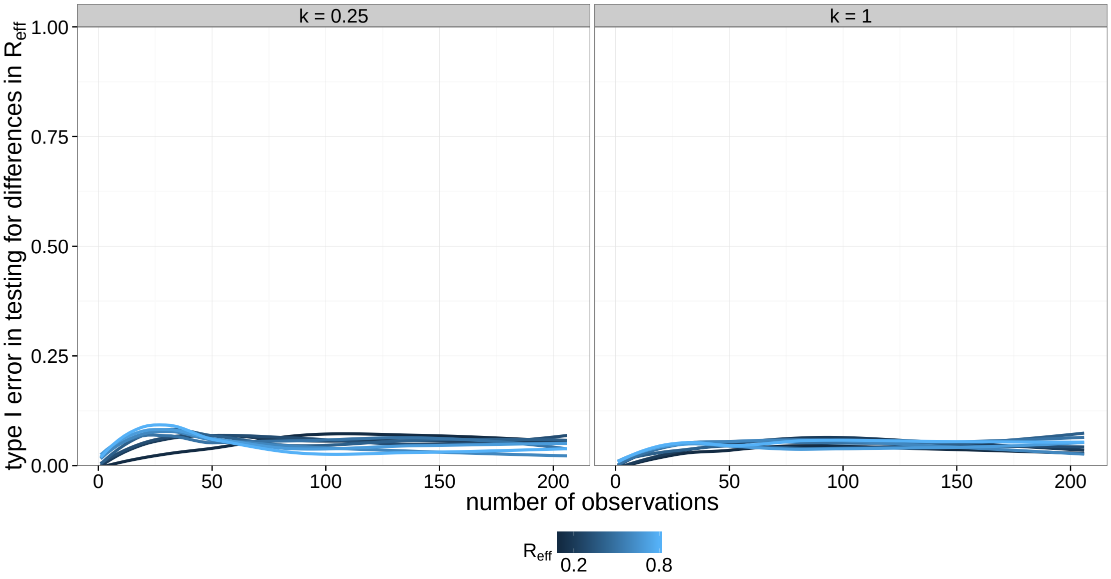

Figure S2: **Type I error.** Results from comparing two sets of simulations of chain size distributions that had the same true value of R_{eff}. The true value of R_{eff} is indicated by the color bar. The simulations all had $k = 0.25$ (left panel) or $k = 1$ (right panel). The y-axis denotes the type I error, or the proportion of simulation pairs for which a difference in R_{eff} was falsely detected (i.e. the best fitting model had different R_{eff}, or all of them did if there were multiple best-fitting models). The curves have been smoothed to improve legibility.

3 Sensitivity of measles analyses to a single large chain

Here we consider the possibility that the largest transmission chain from Canada (155 cases) is an anomalous data point because it is so much larger than second largest chain (30 cases). A conservative approach for addressing this possibility is to exclude the large chain from the analysis. When this is done, we no longer find a significant difference between R_{eff} for United States and Canada (Table 1). The inferred value of R_{eff} for the United States increases only from 0.51 to 0.53 (the small difference occurring because the best model now has a single value of R_{eff}), but the inferred value of R_{eff} for Canada decreases substantially from 0.82 to 0.53.

Given that this approach is expected to make it more difficult to identify true differences in R_{eff}, we performed a parametric bootstrap analysis of the probability to detect a true difference in R_{eff} after the largest chain in the simulated data set has been removed. The parameters for bootstrap simulations were based on estimates of the four parameters in the two R_{eff}, two k model applied to the full measles data set. When matched for the number of chains in the data from the United States and Canada, we found a probability of 0.27 for detecting the estimated difference in R_{eff}. In comparison, when the largest chain was included in the analysis, the parametric bootstrap probability for detecting a change in R_{eff} is twice as large (i.e. 0.55).

Restrictions	Parameters	R_{USA}	k_{USA}	R_{Canada}	k_{Canada}	Log likelihood	ΔAIC
$R_{\text{USA}} = R_{\text{Canada}}$<br>$k_{\text{USA}} = k_{\text{Canada}} = 1$	1	0.53	1	0.53	1	-251.6	7.3
$R_{\text{USA}} = R_{\text{Canada}}$<br>$k_{\text{USA}} = k_{\text{Canada}}$	2	0.53	0.30	0.53	0.30	-246.9	0.0
$k_{\text{USA}} = k_{\text{Canada}} = 1$	2	0.51	1	0.60	1	-251.2	8.5
$R_{\text{USA}} = R_{\text{Canada}}$	3	0.53	0.32	0.53	0.26	-246.9	1.9
$k_{\text{USA}} = k_{\text{Canada}}$	3	0.51	0.31	0.60	0.31	-246.7	1.5
None	4	0.51	0.32	0.60	0.27	-246.7	3.5

Table 1: **Inference results for comparing the transmissibility of measles in the United States (1997–1999) and Canada (1998–2001) when the largest chain is removed.** The layout is analogous to Table 1 of the main text.

4 Adjusting for imperfect observation of monkeypox transmission

Here we assume that each monkeypox case has an independent and identical probability, p_{o}, of activating surveillance. It is also assumed that as long as one case in a cluster activates surveillance, then all cases in the cluster are observed [16]. However, if no cases in a cluster activate surveillance, then no cases in the cluster are observed. This observation model implies that the observed average size of the clusters will be larger than the true average, because the smallest clusters are the least likely to be seen [15, 16]. However, it also favors observation of secondary cases over primary cases because a cluster of size one (i.e. the least likely cluster size to be observed) always contains no secondary cases.

According to our models of animal-to-human and human-to-human transmission for monkeypox, the true probability of a cluster of size j that has m primary cases is:

$$l^C_{1\to m\to j}(R_{\text{a}\to\text{h}}, k_{\text{a}\to\text{h}}, R_{\text{eff}}, k) = l^P_m(R_{\text{a}\to\text{h}}, k_{\text{a}\to\text{h}}) \cdot l^C_{m\to j}(R_{\text{eff}}, k). \tag{10}$$

Summing over possible numbers of primary cases, this relation provides the overall true probability that an animal point source results in a cluster of size j,

$$l^C_{1\to\to j}(R_{\text{a}\to\text{h}}, k_{\text{a}\to\text{h}}, R_{\text{eff}}, k) = \sum_{m=1}^{j} l^C_{1\to m\to j}(R_{\text{a}\to\text{h}}, k_{\text{a}\to\text{h}}, R_{\text{eff}}, k). \tag{11}$$

The probability of not observing a particular cluster of size j is the probability that none of the cases activate surveillance, $(1-p_{\text{o}})^j$. Thus the overall probability that a randomly chosen cluster is

Restrictions	Parameters	$R_{\mathrm{a}\to\mathrm{h}}$	$k_{\mathrm{a}\to\mathrm{h}}$	R_{eff}	k_{eff}	Log likelihood	ΔAIC
$R_{\mathrm{a}\to\mathrm{h}} = R_{\mathrm{eff}}, k_{\mathrm{a}\to\mathrm{h}} = k_{\mathrm{eff}} = 1$	1	0.2	1	0.2	1	-177.7	7.9
$R_{\mathrm{a}\to\mathrm{h}} = R_{\mathrm{eff}}, k_{\mathrm{a}\to\mathrm{h}} = k_{\mathrm{eff}}$	2	0.2	2	0.2	2	-177.2	8.8
$k_{\mathrm{a}\to\mathrm{h}} = k_{\mathrm{eff}} = 1$	2	0.1	1	0.2	1	-174.9	4.3
$\boldsymbol{R_{\mathrm{a}\to\mathrm{h}} = R_{\mathrm{eff}}}$	3	0.2	6.2	0.2	0.2	-171.9	0.2
$\boldsymbol{k_{\mathrm{a}\to\mathrm{h}} = k_{\mathrm{eff}}}$	3	0	0.2	0.2	0.2	-171.8	0.0
None	4	0.1	1.1	0.2	0.2	-171.7	2.0

Table 2: **Inference results for comparing animal-to-human and human-to-human offspring distributions for human monkeypox in the Democratic Republic of Congo, 1981–1984 when $p_o = 0.5$.** The layout is analogous to Table 5 of the main text.

Restrictions	Parameters	$R_{\mathrm{a}\to\mathrm{h}}$	$k_{\mathrm{a}\to\mathrm{h}}$	R_{eff}	k_{eff}	Log likelihood	ΔAIC
$R_{\mathrm{a}\to\mathrm{h}} = R_{\mathrm{eff}}, k_{\mathrm{a}\to\mathrm{h}} = k_{\mathrm{eff}} = 1$	1	0.1	1	0.1	1	-178.4	10.6
$R_{\mathrm{a}\to\mathrm{h}} = R_{\mathrm{eff}}, k_{\mathrm{a}\to\mathrm{h}} = k_{\mathrm{eff}}$	2	0.2	1.9	0.2	1.9	-177.9	11.5
$k_{\mathrm{a}\to\mathrm{h}} = k_{\mathrm{eff}} = 1$	2	0.1	1	0.2	1	-175.3	6.5
$\boldsymbol{R_{\mathrm{a}\to\mathrm{h}} = R_{\mathrm{eff}}}$	3	0.1	3	0.1	0.1	-171.2	0.2
$\boldsymbol{k_{\mathrm{a}\to\mathrm{h}} = k_{\mathrm{eff}}}$	3	0	0.2	0.1	0.2	-171.1	0.0
None	4	0.1	1.6	0.1	0.1	-171.1	2.0

Table 3: **Inference results for comparing animal-to-human and human-to-human offspring distributions for human monkeypox in the Democratic Republic of Congo, 1981–1984 when $p_o = 0.1$.** The layout is analogous to Table 5 of the main text.

unobserved is,

$$p^{C}_{\mathrm{unobs}}(R_{\mathrm{a}\to\mathrm{h}}, k_{\mathrm{a}\to\mathrm{h}}, R_{\mathrm{eff}}, k, p_{\mathrm{o}}) = \sum_{j=1}^{\infty} l^{C}_{1\to\to j}(R_{\mathrm{a}\to\mathrm{h}}, k_{\mathrm{a}\to\mathrm{h}}, R_{\mathrm{eff}}, k) \cdot (1-p_{\mathrm{o}})^{j}. \tag{12}$$

The probability of observing a cluster of size j with m primary infections is then the true probability of this type of cluster occurring times the probability that this cluster will be observed, normalized by the overall probability of a cluster being observed.

$$l^{C}_{\mathrm{obs}:1\to m\to j}(R_{\mathrm{a}\to\mathrm{h}}, k_{\mathrm{a}\to\mathrm{h}}, R_{\mathrm{eff}}, k, p_{\mathrm{o}}) = \frac{l^{C}_{1\to m\to j}(R_{\mathrm{a}\to\mathrm{h}}, k_{\mathrm{a}\to\mathrm{h}}, R_{\mathrm{eff}}, k) \cdot \left(1-(1-p_{\mathrm{o}})^{j}\right)}{1 - p^{C}_{\mathrm{unobs}}(R_{\mathrm{a}\to\mathrm{h}}, k_{\mathrm{a}\to\mathrm{h}}, R_{\mathrm{eff}}, k, p_{\mathrm{o}})}. \tag{13}$$

When we set p_o equal to 0.5 or 0.1, as arbitrarily chosen example values, we find that the preferred model remains $R_{\mathrm{a}\to\mathrm{h}} = R_{\mathrm{eff}}$ (Tables 2 and 3).

References

[1] Harris TE (2002) The Theory of Branching Processes. Toronto: Dover, 256 pp.

[2] Lange K (2010) Applied Probability. New York: Springer, second edition, 452 pp.

[3] Blumberg S, Lloyd-Smith JO (2013) Inference of R_0 and Transmission Heterogeneity from the Size Distribution of Stuttering Chains. PLoS Computational Biology 9: e1002993.

[4] Dwass M (1969) The total progeny in a branching process and a related random walk. Journal of Applied Probability 6: 682–686.

[5] Lloyd-Smith JO, Schreiber SJ, Kopp PE, Getz WM (2005) Superspreading and the effect of individual variation on disease emergence. Nature 438: 355–9.

[6] Alexander HK, Day T (2010) Risk factors for the evolutionary emergence of pathogens. Journal of the Royal Society, Interface 7: 1455–1474.

[7] Newman MEJ (2002) The spread of epidemic disease on networks. Physical Review E 66: 016128.

[8] Meyers LA, Pourbohloul B, Newman MEJ, Skowronski DM, Brunham RC (2005) Network theory and SARS: predicting outbreak diversity. Journal of theoretical biology 232: 71–81.

[9] Cauchemez S, Fraser C, Van Kerkhove MD, Donnelly CA, Riley S, et al. (2014) Middle east respiratory syndrome coronavirus: quantification of the extent of the epidemic, surveillance biases, and transmissibility. The Lancet infectious diseases 14: 50–56.

[10] Gay NJ, De Serres G, Farrington CP, Redd SB, J M (2004) Assessment of the status of measles elimination from reported outbreaks: United States, 1997-1999. The Journal of Infectious Diseases 189 Suppl: S36-S42.

[11] King A, Varughese P, De Serres G, Tipples GA, Waters J, et al. (2004) Measles elimination in Canada. The Journal of Infectious Diseases 189 Suppl: S236–42.

[12] Fenner F, Henderson DA, Arita I, Jezek Z, Ladnyi ID (1988) Smallpox and its Eradication. Geneva: World Health Organization, 1460 pp.

[13] Jezek Z, Grab B, Dixon H (1987) Stochastic model for interhuman spread of monkeypox. American Journal of Epidemiology 126: 1082–92.

[14] Fine PE, Jezek Z, Grab B, Dixon H (1988) The transmission potential of monkeypox virus in human populations. International Journal of Epidemiology 17: 643–50.

[15] Blumberg S, Lloyd-Smith JO (2013) Comparing methods for estimating R_0 from the size distribution of subcritical transmission chains. Epidemics 5: 131–145.

[16] Ferguson NM, Fraser C, Donnelly CA, Ghani AC, Anderson RM (2004) Public health risk from the avian H5N1 influenza epidemic. Science 304: 1–5.
