# Supplementary material for: Detecting Differential Transmissibilities That Affect the Size of Self-Limited Outbreaks
Source: PLoS Pathog. 2014 Oct 30;10(10):e1004452. doi: 10.1371/journal.ppat.1004452 (PMC4214794; doi:10.1371/journal.ppat.1004452)
Supplement: Text S2 — Data supplement. Provides tables and references for all the data used in the analyses. (PDF) [file ppat.1004452.s002.pdf]

Data: Detecting differential transmissibilities that affect the size of self-limited outbreaks

Seth Blumberg, Sebastian Funk, Juliet R. C. Pulliam

August 31, 2014

References

[1] Cauchemez S, Fraser C, Van Kerkhove MD, Donnelly CA, Riley S, et al. (2014) Middle east respiratory syndrome coronavirus: quantification of the extent of the epidemic, surveillance biases, and transmissibility. The Lancet infectious diseases 14: 50–56.

[2] Gay NJ, De Serres G, Farrington CP, Redd SB, J M (2004) Assessment of the status of measles elimination from reported outbreaks: United States, 1997-1999. The Journal of Infectious Diseases 189 Suppl: S36-S42.

[3] King A, Varughese P, De Serres G, Tipples GA, Waters J, et al. (2004) Measles elimination in Canada. The Journal of Infectious Diseases 189 Suppl: S236–42.

[4] Fenner F, Henderson DA, Arita I, Jezek Z, Ladnyi ID (1988) Smallpox and its Eradication. Geneva: World Health Organization, 1460 pp.

[5] Jezek Z, Grab B, Dixon H (1987) Stochastic model for interhuman spread of monkeypox. American Journal of Epidemiology 126: 1082–92.

[6] Fine PE, Jezek Z, Grab B, Dixon H (1988) The transmission potential of monkeypox virus in human populations. International Journal of Epidemiology 17: 643–50.

Chain size	Number of chains before June 1, 2013	Number of chains after June 1, 2013
1	11	16
2	1	0
3	3	1
4	2	1
5	1	1
7	1	0
7	1	0
26	1	0

Table 1: **Distribution of observed transmission chains of MERS-CoV in the Arabian Peninsula (From Table S4 of Cauchemez et al) [1] used in Section 3.1 of the main text.**

Cluster size	Number of clusters<br>United States ('97-'99)	Number of clusters<br>Canada ('98-'01)
1	122	35
2	13	5
3	10	3
4	6	1
5	5	0
6	2	1
8	2	1
9	1	0
11	1	0
13	1	0
15	1	0
17	0	1
30	0	1
33	1	0
155	0	1

Table 2: **Distribution of observed infection clusters of measles in the United States [2] and Canada [3] used in Section 3.2 of the main text.** All clusters have a single primary case, except for one chain of size six in the United States that had two primaries

Transmission	Primary cases	Secondary cases
$1 \to 0$	14	7
$1 \to 1$	7	1
$1 \to 2$	4	0
$1 \to 3$	1	0
$1 \to 4$	1	0
$1 \to 6$	0	1
$1 \to 10$	2	0
$1 \to 11$	1	0
$1 \to 13$	1	0
$1 \to 17$	1	0
$1 \to 18$	0	1
$1 \to 19$	1	0
$1 \to 2$	0	2
$2 \to 0$	0	5
$2 \to 1$	0	2
$2 \to 2$	0	1
$2 \to 4$	0	1
$3 \to 0$	0	6
$3 \to 11$	1	0
$3 \to 20$	0	1
$4 \to 10$	0	1
$4 \to 26$	0	1
$6 \to 3$	0	2
$6 \to 18$	0	1
$7 \to 1$	0	1
$1 \to 03$	0	1
$10 \to 6$	0	1
$10 \to 7$	0	1
$11 \to 19$	0	1
$11 \to 140$	0	1
$13 \to 3$	0	1
$17 \to 2$	0	1
$18 \to 1$	0	1
$18 \to 2$	0	1
$19 \to 0$	0	1
$19 \to 23$	0	1
$20 \to 3$	0	1
$20 \to 6$	0	1
$23 \to 3$	0	1
$24 \to 0$	0	1
$26 \to 44$	0	1
$44 \to 20$	0	1
$140 \to 24$	0	1

Table 3: **Generation-to-generation observed spread of smallpox in Europe prior to smallpox eradication (From Table 23.4 of Fenner et al. [4] used in Section 3.3 of the main text.** Each row represents a single generation of spread (i.e. $3 \to 11$ refers to 3 cases in one generation leading to 11 cases in the following generation). The primary cases column refer to the number of primary cases that exhibit the transmission pattern of the leftmost column. The secondary cases column provides analogous data for instances where secondary cases are the source of transmission.

Transmission	Primary cases	Secondary cases
$1 \to 0$	114	50
$1 \to 1$	14	7
$1 \to 2$	6	2
$1 \to 3$	1	1
$1 \to 5$	1	0
$2 \to 1$	0	1
$2 \to 2$	3	0
$2 \to 4$	1	0
$3 \to 3$	1	0

Table 4: **Generation-to-generation spread of observed monkeypox in the Democratic Republic of Congo [5, 6] used in Section 3.4 of the main text.** Each row represents a single generation of spread (i.e. $3 \to 3$ refers to 3 cases in one generation leading to 3 cases in the following generation). The primary cases column refer to the number of primary cases that exhibit the transmission pattern of the leftmost column. The secondary cases column provides analogous data for instances where secondary cases are the source of transmission.

Cluster transmission	Number clusters
$1 \to 1$	84
$1 \to 2$	7
$1 \to 3$	9
$1 \to 4$	2
$1 \to 5$	2
$1 \to 6$	2
$2 \to 2$	12
$2 \to 4$	3
$2 \to 6$	1
$3 \to 3$	2
$3 \to 6$	1

Table 5: **Data on the observed number of primary cases and sizes of infection clusters for monkeypox in the Democratic Republic of Congo [5, 6] used in Section 3.5 of the main text.** Each row represents a single infection cluster (i.e. $2 \to 4$ refers to an infection cluster having 4 cases, 2 of which are primary).
